# Eprinomectin from a sustained release formulation adversely affected dung breeding insects

**DOI:** 10.1371/journal.pone.0201074

**Published:** 2018-08-06

**Authors:** Christine C. Nieman, Kevin D. Floate, Rolf-Alexander Düring, Andre P. Heinrich, Daniel K. Young, Daniel M. Schaefer

**Affiliations:** 1 Department of Animal Sciences, University of Wisconsin, Madison, Wisconsin, United States of America; 2 Agriculture and Agri-Food Canada, Lethbridge, Alberta, Canada; 3 Institute of Soil Science and Soil Conservation, Justus Liebig University, Giessen, Germany; 4 Department of Entomology, University of Wisconsin, Madison, Wisconsin, United States of America; Montana State University Bozeman, UNITED STATES

## Abstract

The insecticidal activity of parasiticide residues in dung of cattle treated with a sustained release eprinomectin formulation was examined, and an improved eprinomectin dung residue extraction method is presented. Emergent insect abundance and richness were significantly reduced in all post-treatment intervals (7, 14, 28, 56, 84, 112, and 140 d), relative to pre-treatment. Emergent insect diversity was reduced for between 84 and 112 d post-treatment. Collembola were not affected by residues. Chemical analyses subsequently documented residues of eprinomectin in dung of each collection period post-treatment at levels expected based on previously reported excretion profiles for this product. Cattle subcutaneously injected with this product excreted residues that reduced dung-breeding insect emergence for 5 mo post-treatment. The consequences of these long-term non-target effects to pasture ecosystems are not known.

## Introduction

Avermectins (e.g., doramectin, ivermectin, eprinomectin) are veterinary parasiticides used on livestock. Avermectins are minimally metabolized in livestock, are fecally-excreted and have insecticidal activity [1]. Application methods include use of an oral paste, subcutaneous injection, topical application, and sustained release boluses administered orally to the rumen. Eprinomectin from topical application had lower maximum plasma and dung concentrations and longer mean retention times in plasma and dung compared to eprinomectin administered by subcutaneous injection [2]. The prolonged dung excretion of topical avermectin formulations reduced adult insect emergence in dung of treated cattle for up to 84 d [3] and 112 d [4] post-application. Residues in dung of cattle treated with ivermectin applied in a sustained release bolus killed dung beetle larvae for up to 143 d [5].

The insecticidal activity of fecal residues can have both beneficial and adverse effects on pasture ecology. Residues can reduce populations of pestiferous flies that breed in dung; i.e., *Haematobia irritans* (Linnaeus) [6–7] and *Musca autumnalis* De Geer [8]. Conversely, residues may reduce populations of benign or beneficial species [3,6,9] including predaceous and parasitoid species that feed on eggs and larvae of pest species [10–12] and dung beetles (Hydrophilidae, Ptiliidae, Scarabaeidae) that disrupt the pat creating a less suitable breeding site for pest fly species and freeing the pasture surface for the growth of new vegetation [13]. Some species of dung beetles also bury parts of the dung pat, resulting in aeration of the soil, increased water permeability, and transfer of nutrients into the soil, as reviewed by Nichols et al [14].

LongRange^®^ (Merial Ltd.), a slow release eprinomectin-containing product, became commercially available in 2012. The product is injected subcutaneously and contains 50 mg mL^-1^ of eprinomectin in a cosolvent system of N-methyl-2-pyrrolidone (30% v/v) and triacetin, along with 50 mg mL^-1^ of poly-lactide-coglycolic-acid. This biodegradable matrix solidifies in vivo to form an in situ gel, which enables a delayed release of eprinomectin. The extended release of eprinomectin occurs in a bimodal fashion, characterized by peak concentrations of eprinomectin in plasma of cattle within a few days post-treatment followed by a second smaller peak around day 90 [15]. Based on this profile, we hypothesized that cattle treated with LongRange would excrete eprinomectin in dung for at least 140 d at levels sufficient to reduce insect activity in dung. The current study was undertaken to test this hypothesis and, to our knowledge, is the first study to report the non-target effects of eprinomectin in dung from cattle treated with LongRange.

## Materials and methods

The experiment was conducted at the Arlington Agricultural Research Station in Arlington, Wisconsin (43.3380°N, 89.3804°W). LongRange^®^ was administered as directed on the manufacturer’s label, with a dosage of 1 mg of eprinomectin per kg of body weight (BW), injected subcutaneously in front of the shoulder.

Holstein steers (N = 10, BW 374 ± 40 kg (mean ± SD)), housed together in one pen for the duration of the dung collection, were treated with LongRange in October 2014. Cattle were maintained on a constant diet of 40% grass hay, 40% alfalfa silage, and 20% cracked corn, with access to free choice salt for the entire period of dung collection. Feces were collected before treatment, to serve as the control (day 0), and 7, 14, 28, 56, 84, 112, and 140 d after dosing. Fecal collections from approximately 8 pats were taken at 0800 h from the pen floor less than 2 h after defecation over 3 d, a day prior, day of, and a day after the exact collection day, with the exception of day 0, in which feces were collected 3 d prior to treatment day 1. Feces collected during each 3-day span were homogenized and stored in garbage bags in airtight pails (18.9 L) that were frozen at -18°C until the following spring. Feces were thawed in spring and thoroughly mixed in the garbage bag to reduce potential variation among pats.

Cattle were checked daily in accordance with the standard operating procedure of the Arlington Beef Center. The research protocol (A01420) was approved by the College of Agricultural and Life Sciences Animal Care and Use Committee.

### Dung pat exposure

Pats of standard size and shape (500 g each, 12 pats per sampling day) were formed using a circular plastic mold 13.9 cm in diameter. Pats were placed on aluminum plates 22.9 cm in diameter with approximately 2.54 cm of damp sand between the plate surface and the artificial pat. Holes of 4 mm in diameter were cut into the bottom of the plate for drainage. Plates were placed in a pasture previously grazed by cattle and adjacent to grazing cattle. A total of 96 (8 collection dates x 12 replicates) pats were placed in a 9.5 by 18.2 m grid with three columns. Pats from each treatment were randomly distributed in a column, resulting in 4 replicates per treatment in each column. Pats were covered with chicken wire to prevent disturbance from birds. Columns were separated by approximately 3 m and pats within a column were separated by approximately 0.57 m. Pats were exposed for 5 d (23–28 June 2015). Precipitation during the exposure period was minimal (total 0.16 cm), and the average, minimum, and maximum temperatures were 19.7°C, 14.0°C, and 24.9°C, respectively.

### Insect emergence

Pats were then placed in emergence cages and transported to the Livestock Laboratory on the University of Wisconsin-Madison campus, and kept in a temperature (22°C) controlled room that was continuously illuminated. Despite the use of chicken wire, some pats across all treatments were disrupted by wildlife. To remove this disruption as a confounding factor, these pats (n = 10) were excluded from further consideration. Emergence cages were 11 L pails fitted with a fine-mesh tulle, secured to the pail with a silicone band to prevent the escape of insects. Adult insects were removed from emergence cages weekly, until emergence stopped at approximately 10 wk. Chlorinated tap water (60 mL) was evenly dispensed onto each dung pat every 2 wk to reduce desiccation of developing insects. Dung organic matter is assumed to have quenched the biocidal activity of chlorine. Specimens collected weekly were stored in vials containing 70% ethanol.

Because our objective was to test the effect of excreted eprinomectin on insects developing in dung pats from egg-to-adult, we excluded ‘colonizers’ from consideration [3]. This was only a concern for beetles, for which adults colonized pats in the field and were present in or beneath the dung at the time the pats were placed in emergence cages. These colonizing beetles were identified by their presence in cages in the first 4 wk after pats were returned from the field; i.e., before they could have completed egg-to-adult development [3]. In contrast, adult flies will vacate a pat that is disturbed. Hence, all adult flies recovered in cages were assumed to have developed in pats and were retained for analyses.

### Statistical analyses

Community-level analyses were performed by measuring the abundance, richness, and diversity for all taxa combined (insect Families and Collembola). The parameters were defined as follows: abundance = the total number of individuals emerged; richness = the total number of insect Families and Order Collembola; and diversity = Hill’s ¹D diversity (exp [Shannon Index H’]) [16]. Abundance analyses were then performed on individual taxa.

To reduce the likelihood of type II errors, analyses were limited to taxa represented by at least 10 individuals or when the frequency was at least one individual recorded per day 0 pat. This threshold eliminated many taxa for which analyses were unlikely to detect eprinomectin effects. All analyses on individual taxa were performed at the level of Family, except for Collembola, which were identified only to Order.

Data were non-normally distributed (Shapiro-Wilks test, *P* < 0.05) and exhibited extreme heteroscedasticity across treatments. Therefore, data were rank-transformed prior to use of ANOVA tests [17]. For the community level tests, abundance, richness, or diversity was set as the dependent variable with sampling day as the fixed factor. When a treatment effect was detected (*P* < 0.05), a post hoc Dunnett’s test (1-sided) was performed to test for differences between the pre-treatment sample (day 0) and each subsequent sampling day. For tests on individual taxa, insect abundance was set as the dependent variable with sampling day as the fixed factor. Similarly, data for community level tests were non-normally distributed and were rank transformed. A post hoc Dunnett’s test (1-sided) was performed on significant treatment effects (*P* < 0.05) between the pre-treatment sample (day 0) and each later sampling day. To ensure that coleopteran colonizers (F-0 generation) were not unequally attracted among sampling days, an ANOVA was conducted to compare insect numbers across sampling days.

### Chemical analysis

To better establish a link between eprinomectin excretion and insect activity, eprinomectin concentrations in dung were measured for each sampling day (0, 7, 14, 28, 56, 84, 112, and 140 d). For these measurements, an aliquot of dung was set aside at time of collection and frozen at -18°C. Following a storage duration of 26–31 mo, the dung was thawed and residues were extracted from 3–5 g samples. Details of the underlying extraction process and residue measurement with HPLC-FLD were based on Wohde et al [18] and Kozuh Erzen et al [19] and are described briefly. A detailed description is included in the appendix.

Standard solutions for calibration were prepared in acetonitrile (≥99.9% HiPerSolv CHROMANORM, gradient grade for HPLC; VWR International) using eprinomectin (purity 94.3%, CA13187000). To assess any matrix effects, ivermectin (purity 96%, CA14488000) was chosen as the internal standard since the analytical method for its determination is well established, it also belongs to the avermectin family of compounds, and it can be well-separated from eprinomectin during chromatography with a C18-Column. Purity of both standards were determined by Dr. Ehrenstorfer GmbH (Augsburg, Germany).

Water content was determined in duplicate according to DIN EN 15934:2012–11. For each sampling day, 5 g of thawed and homogenized dung were dried for 24 h at 105°C. Fresh dung samples had a mean water content of approximately 79.5% ranging from 76.8% to 84.2%. Later on, samples were not rehydrated. For the extraction, 25 mL acetonitrile and 250 μL ivermectin working solution (2000 μg L^-1^) were added to 3 g of homogenized moist dung. Further extraction steps complied with the method described by Wohde et al [18] and also included the solid phase extraction step. Deviations from this methodology are mentioned in S1 Methods.

Area under the eprinomectin dung excretion curve was calculated using the Miscellaneous Esoteric Statistical Scripts (MESS) package (https://cran.r-project.org/web/packages/MESS/MESS.pdf), which can be used via the statistical software R [20]. For calculating the area under the curve, a spline interpolation was used and the numerical integral was calculated for each mathematical function.

## Results and discussion

A total of 4810 insects were recovered from emergence cages, representing seven Orders spanning 32 Families. After removal of coleopteran colonizers and taxa with insufficient individuals for analyses, 228 collembolans, 867 coleopterans (four Families), and 1108 dipterans (six Families) were retained for analyses. Fecal residues of avermectins may attract, repel or have no effect on the colonization of dung by coprophilous insects [21–27]. Because the number of coleopteran colonizers did not differ by sampling day (P = 0.43), fecal residues in the current study were assumed not to have affected colonization across treatment groups. The consequences of altered colonization associated with avermectin residues are discussed in more detail by Floate [25].

### Community analyses

**Fig 1:**
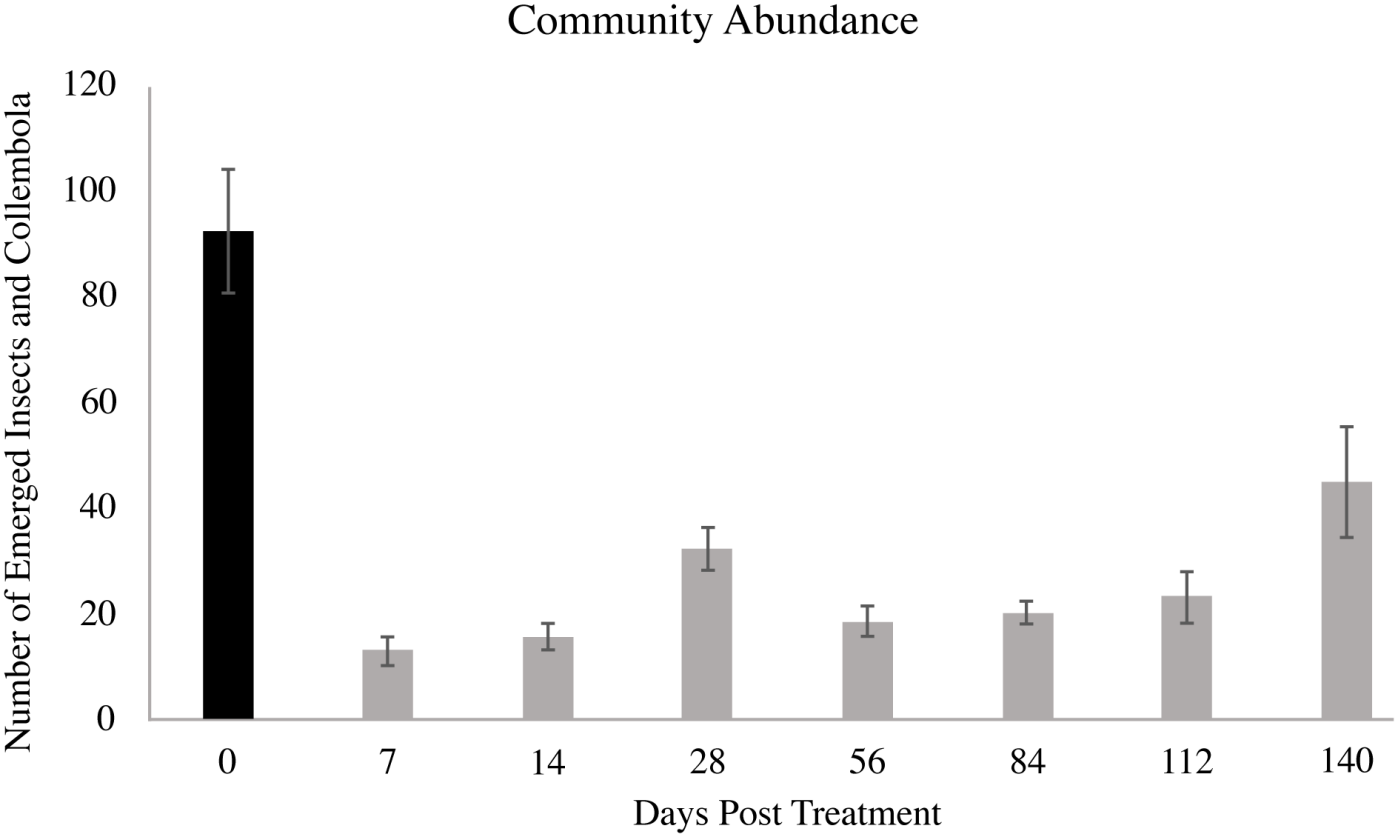
Total insect and Collembola emergence. The total number of insects and Collembola that emerged from dung (500 g) deposited by cattle before (day 0) and 7, 14, 28, 56, 84, 112, and 140 d after subcutaneous injection of LongRange eprinomectin (1 mg eprinomectin kg^-1^ body weight). Error bars denote the respective standard error of the mean. Statistical analysis compared the control (day 0) to each post-injection sampling day. Reported means and standard errors are from un-ranked data, while significance was based on analyses of rank-transformed data. Grey bars indicate values that are different (*P* < 0.05) than the control (day 0).

**Fig 2:**
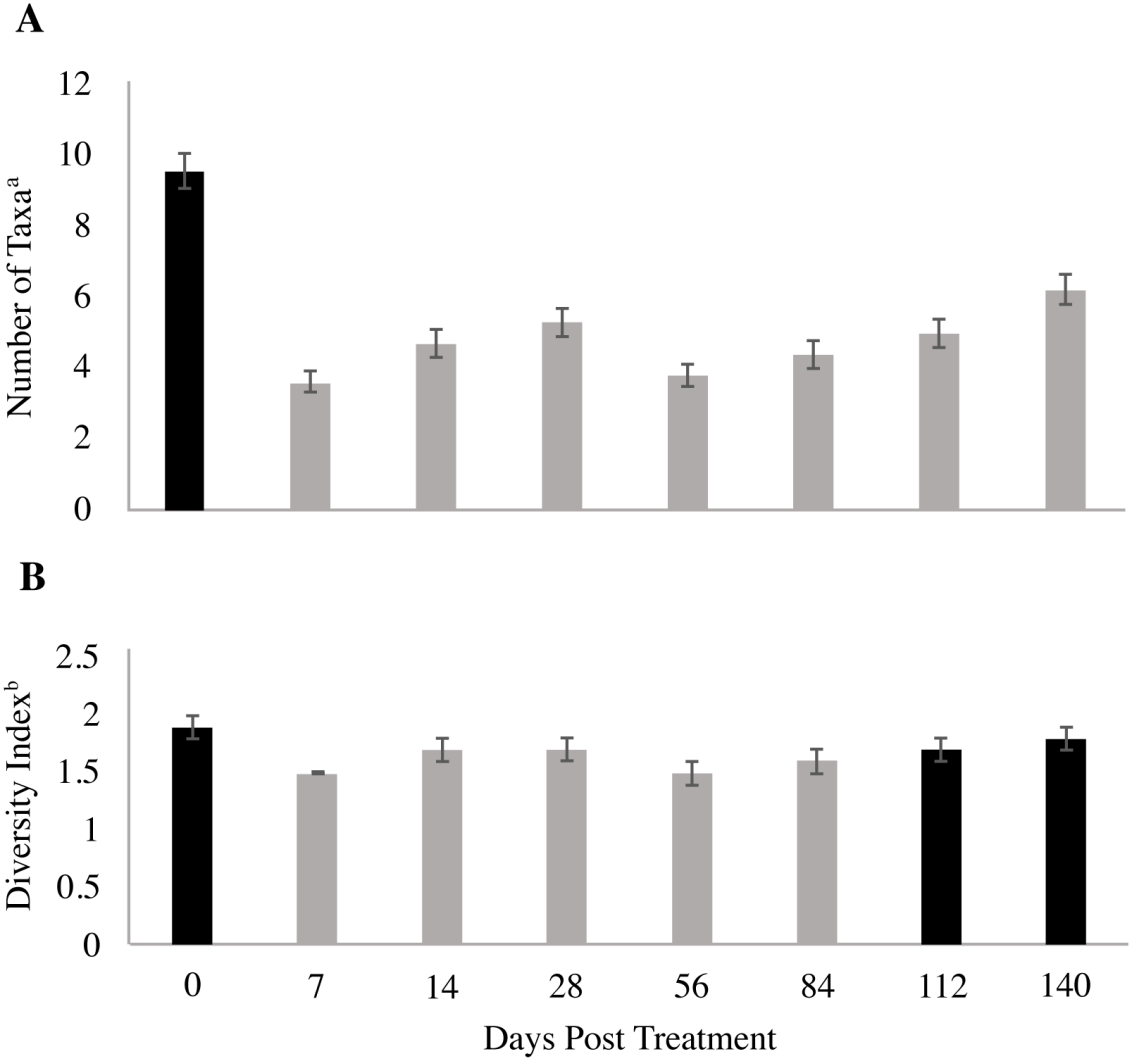
Total insect and Collembola community richness and diversity index. Community richness (**A**) and community diversity index (**B**) for insect communities and Collembola that emerged from dung (500 g) deposited by cattle before (day 0) and 7, 14, 28, 56, 84, 112, and 140 d after subcutaneous injection of LongRange eprinomectin (1 mg eprinomectin kg^-1^ body weight). Error bars denote the respective standard error of the mean. Statistical analysis compared the control (day 0) to each post-injection sampling day. Reported means and standard errors are from un-ranked data, while significance was based on analyses of rank-transformed data. Grey bars indicate values that are different (*P* < 0.05) than the control (day 0). ^a^Number of taxa is the total number of Families plus the Order Collembola. ^b^Diversity index is Hill’s ^1^D diversity (exp [Shannon Index H’]).

**Fig 3:**
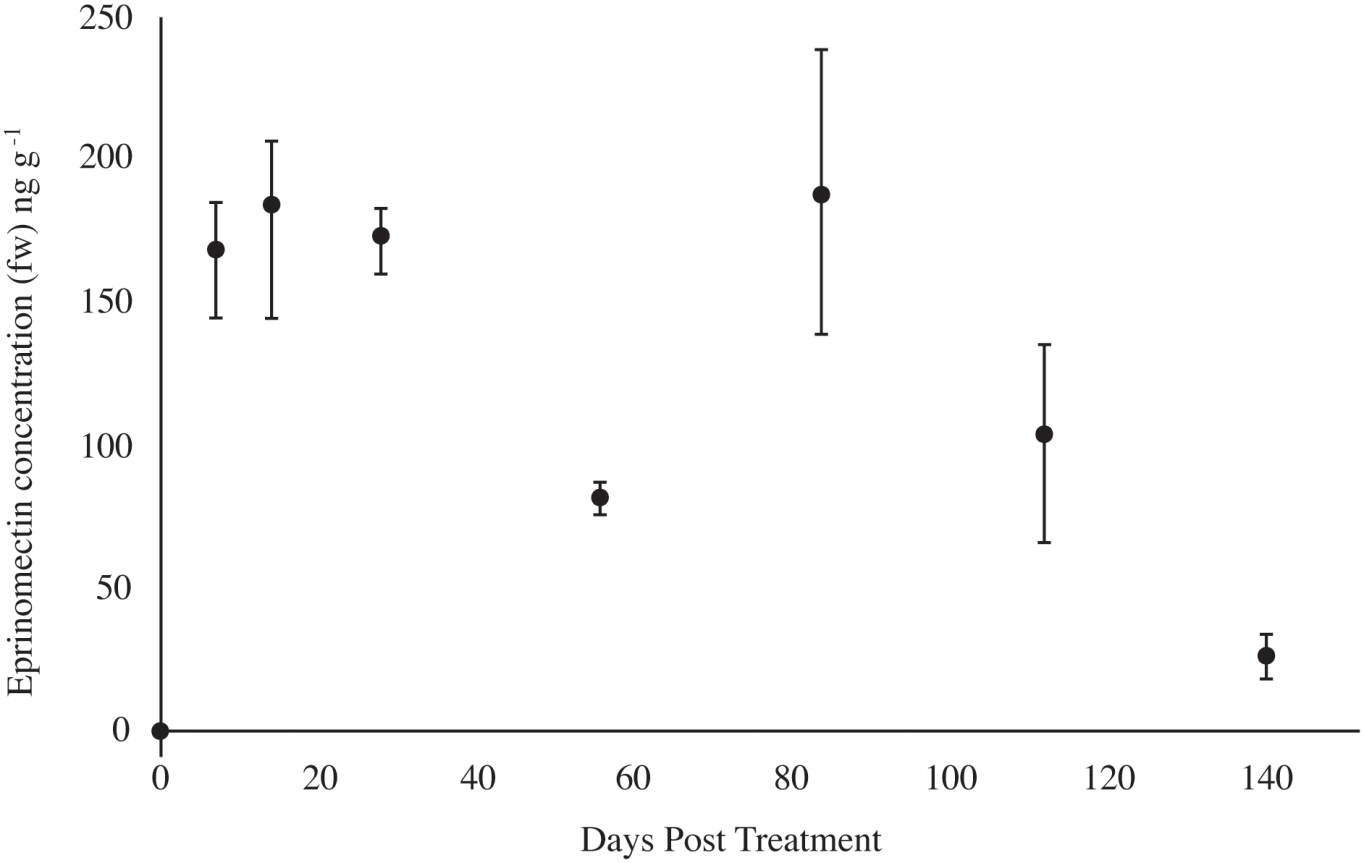
Eprinomectin concentration in cattle dung. Excretion profile of eprinomectin in dung (fresh weight, fw; mean ± minimum and maximum values of 3 replicates; n = 8) of cattle collected before (day 0) and 7, 14, 28, 56, 84, 112 and 140 d after LongRange treatment.

Use of LongRange was associated with significant reductions in insect abundance (Fig 1) and richness (Fig 2) at all sampling days post-application, and with significant reductions in diversity (Fig 2) up to and including day 84 post-application. Dung collected from day 7 had the lowest values for abundance and richness, while the lowest values for diversity occurred at days 7 and 56. The second highest values for abundance and richness occurred at day 140. Diversity values for days 112 and 140 did not differ from day 0, possibly due to the declining dung eprinomectin concentration after day 84 (Fig 3).

### Taxon analyses

Analyses on individual taxa (Table 1) were performed on Collembola and for Families of both Coleoptera (Hydrophilidae, Ptiliidae, Scarabaeidae, Staphylinidae) and Diptera (Calliphoridae, Fanniidae, Muscidae, Psychodidae, Sphaeroceridae, Stratiomyidae).

**Table 1:** Number of insects in each family and Collembola emerging from dung of treated cattle^a^.

ORDER Family	Day 0^b^ *n* = 12	Day 7 *n* = 11	Day 14 *n* = 10	Day 28 *n* = 10	Day 56 *n* = 10	Day 84 *n* = 12	Day 112 *n* = 10	Day 140 *n* = 11	*P* value Ranked	Duration of effect^c^
COLLEMBOLA	3.8 ± 0.7	2.4 ± 0.6	3.2 ± 0.9	4.1 ± 1.6	1.7 ± 0.5	1.8 ± 0.5	2.2 ± 0.5	2.1 ± 0.6	0.370	no effect
COLEOPTERA										
Hydrophilidae	6.1 ± 1.3A	0.1 ± 0.1B	0.5 ± 0.2B	1.9 ± 0.6B	0.5 ± 0.2B	0.1 ± 0.1B	1.5 ± 0.9B	1.3 ± 0.3B	<0.001	≥ 140 d
Ptiliidae	1.1 ± 0.3A	0.0 ± 0.0B	0.0 ± 0.0B	0.0 ± 0.0B	0.0 ± 0.0B	0.0 ± 0.0B	0.0 ± 0.0B	0.0 ± 0.0B	<0.001	≥ 140 d
Scarabaeidae	1.2 ± 0.7A	0.0 ± 0.0B	0.0 ± 0.0B	0.0 ± 0.0B	0.0 ± 0.0B	0.0 ± 0.0B	0.7 ± 0.2A	6.9 ± 2.7B	<0.001	112–140 d
Staphylinidae	32.5 ± 3.8A	0.0 ± 0.0B	0.0 ± 0.0B	0.0 ± 0.0B	0.0 ± 0.0B	0.0 ± 0.0B	10.7 ± 2.7B	11.5 ± 1.6B	<0.001	≥ 140 d
DIPTERA										
Calliphoridae	1.3 ± 0.6A	0.0 ± 0.0B	0.0 ± 0.0B	0.0 ± 0.0B	0.0 ± 0.0B	0.0 ± 0.0B	0.0 ± 0.0B	0.0 ± 0.0B	<0.001	≥ 140 d
Fanniidae	24.7 ± 11.2A	0.0 ± 0.0B	0.0 ± 0.0B	0.0 ± 0.0B	0.0 ± 0.0B	0.0 ± 0.0B	0.0 ± 0.0B	0.4 ± 0.2B	<0.001	≥ 140 d
Muscidae	1.5 ± 0.6A	0.0 ± 0.0B	0.0 ± 0.0B	0.0 ± 0.0B	0.0 ± 0.0B	0.0 ± 0.0B	0.0 ± 0.0B	0.0 ± 0.0B	<0.001	≥ 140 d
Psychodidae	2.0 ± 0.6A	0.2 ± 0.2B	0.0 ± 0.0B	0.0 ± 0.0B	0.0 ± 0.0B	0.0 ± 0.0B	0.0 ± 0.0B	0.0 ± 0.0B	<0.001	≥ 140 d
Sphaeroceridae	1.3 ± 0.4A	0.6 ± 0.3A	0.5 ± 0.3A	0.5 ± 0.3A	0.0 ± 0.0B	1.6 ± 0.6A	0.5 ± 0.3A	6.1 ± 1.4B	<0.001	no effect
Stratiomyidae	14.6 ± 4.2A	0.3 ± 0.3B	0.0 ± 0.0B	0.0 ± 0.0B	0.0 ± 0.0B	0.0 ± 0.0B	6.1 ± 2.2B	14.5 ± 9.8A	<0.001	112–140 d

^a^ Numbers of insects (mean ± SE) emerging from dung (500 g) deposited by cattle before (day 0) and 7, 14, 28, 56, 84, 112, and 140 days after subcutaneous application of LongRange eprinomectin (1 mg kg^-1^ body weight).

^b^ Analyses compared the control pre-application (day 0) to each post-application sampling day within the same row. Reported means and standard errors are from un-ranked data, while *P*-values are based on analyses of rank-transformed data. Control pre-application and post-application means with different letters differ (*P* < 0.05).

^c^ Period during which treatment value is significantly less than control.

No effect of LongRange administration was found with respect to Collembola (*P* = 0.37). Previous studies assessing the toxicity of avermectin residues on Collembola produced mixed results, but seem to indicate that residues are unlikely to affect Collembola in field situations (reviewed in Scheffczyk et al. [28]).

For Coleoptera, adult emergences of Hydrophilidae, Ptiliidae, and Staphylinidae were reduced in dung from cattle treated up to and including 140 d prior (*P* < 0.05, Table 1). The sensitivity of these taxa to avermectins has been reported elsewhere. Floate [3] reported reduced adult emergences of Hydrophilidae and Staphylinidae from dung of cattle topically-treated 12 wk previously with ivermectin. Depending upon the species and compound, adult emergence of Ptiliidae and species of Staphylinidae was either unaffected, or was reduced in dung of cattle treated up to 4 wk previously with either doramectin, eprinomectin, or ivermectin [9].

A reduction in adult emergence of Scarabaeidae was detected in dung up to only 84 d post-treatment. This sampling day coincided with a maximum in eprinomectin concentration (188 ng g^-1^), after which eprinomectin concentration decreased (Fig 3) and Scarabaeidae emergence returned to the day 0 level. Reduced adult emergence has been reported for *Calamosternus* (previously *Aphodius*) *granarius* (L.), *Aphodius fimetarius* (L.), and *Planolinellus* (previously *Aphodius*) *vittatus* (Say) developing in dung of cattle treated with ivermectin [3]. Reduced survival has been reported for *Onthophagus taurus* (Schreber) developing in dung of cattle treated up to 14 d previously with a topical application of eprinomectin [29]. Adult emergence of *Caccobius jessoenisis* (Harold) was suppressed in dung of cattle injected with a recommended dose of eprinomectin up to 3 d previously, but not 7 d previously, by which time eprinomectin concentrations declined to approximately 50 ng g^-1^ wet weight [7].

For Diptera, adult emergence was suppressed (*P* < 0.001) for almost all taxa through day 140 after LongRange administration. This finding is consistent with previous studies. In laboratory studies, survival of *Neomyia cornicina* (Fabricius) and *H*. *irritans* was reduced in dung from cattle treated 12 and 14 d previously with a topical application of eprinomectin [7, 29, 90]. Reduced survival of *N*. *cornicina* was detected in dung with eprinomectin concentrations as low as 57 ng g^-1^ wet weight [30]. The exceptions to the Diptera response was Sphaeroceridae, for which a reduction was detected only for d 56, and the Stratiomyidae which were suppressed only to day 112. Given previous reports of sphaerocerid reductions in dung of cattle treated with avermectins ([3, 9, 17, 31], results reported in this study are atypical.

Only ivermectin in the slow release bolus formulation reduces adult insect emergence for a duration equivalent to that reported here for LongRange. In one of the few such studies to test the non-target effects of the slow release ivermectin bolus, larval development of *Aphodius constans* (Duftschmid) was reduced in dung from cattle treated 105 and 143 d previously, compared to the control [5]. These reductions were documented at ivermectin concentrations as low as 38 ng g^-1^ dung wet weight [5].

### Dung eprinomectin concentrations

The double-peak excretion profile of eprinomectin (Fig 3) is consistent with previous reports for LongRange [15]. Eprinomectin concentration increased to 184 ng g^-1^ on day 14, declined to 82 ng g^-1^ at day 56, increased to 188 ng g^-1^ on day 84, and then declined to 27 ng g^-1^ at day 140. Lumaret et al [30] showed reduced survival of *N*. *cornicina* at eprinomectin levels as low as 57 ng g^-1^. Dung eprinomectin in this study exceeded 27 ng g^-1^ for all days except 140 d, and these concentrations suppressed emergence of nearly all Dipteran species, except Sphaeroceridae, and almost all Coleopterans except Scarabaeidae.

Given the steer average BW of 374 kg, eprinomectin dosage of 1 mg kg^-1^ BW, and mean dung concentrations (Fig 3), recovery of eprinomectin in dung was estimated using a spline function. Based on the average detected eprinomectin concentration, the area under the excretion profile was 2537 ng eprinomectin × wk g^-1^ fw dung, or 2.54 mg × wk kg^-1^. Diet dry matter (DM) intake of the steers was estimated to be 0.022 kg diet kg^-1^ BW, growth rate during the trial was estimated to be 1 kg BW gain d^-1^ [32] resulting in average BW for the trial of 444 kg. Indigestible diet DM was estimated to be 30% of diet DM. Indigestible diet DM was converted to dung fw using the average dung DM content (20.5%). This resulted in an estimated dung output of 14.3 kg d^-1^ or 100.1 kg wk^-1^. The average result is that 254 mg of the 374 mg, or 67.9%, of injected eprinomectin has been tallied. Considering the varying BW at treatment day (SD ± 40 kg) and including the minimum and maximum dung concentrations of the triplicate measurements for each sampling day, the total tally ranges from 49.2 to 89.2%. When compared to pharmacokinetics of ivermectin in cattle (Canga et al. 2009), these values indicate a credible analytical recovery range for eprinomectin in dung. Notably, an average of 27.3 ng g^-1^ (fw) could still be detected after 140 d. Thus, additional excretion of eprinomectin and potential release into the environment after the study duration can be expected.

The claim for LongRange is that it provides persistent control of parasites for up to 100 to 150 d (Merial Ltd., 2012). Grazing seasons in northern latitudes of the U.S. have a duration of 150 to 180 d. If cattle are treated with LongRange near the initiation of the grazing season, the first surge of high dung eprinomectin concentration would coincide with the egg-laying period for many of the dung breeding insect species. Three months later, adult insects feeding in dung could again be exposed to the second surge of eprinomectin, resulting in potential lethal or sub-lethal effects that could compromise their overwintering ability.

## Conclusions

Cattle treated with eprinomectin in the LongRange formulation excreted eprinomectin in a bimodal manner with dung concentration maxima near day 14 and 84. At day 140, eprinomectin was detected at 27 ng g^-1^ dung. Insect emergence did not appear to follow a bimodal pattern. Emergence from dung by Hydrophilidae, Ptiliidae, and Staphylinidae was adversely affected through day 140. Scarabaeidae were affected up to day 84. For Diptera families, except Sphaeroceridae, adult insect emergence was suppressed to day 140, though for Stratiomyidae only to day 112. Collembola and Sphaeroceridae were not importantly affected by LongRange injection.

For those families affected by fecal eprinomectin, suppression of their emergence from dung occurred through day 140 after LongRange administration. This duration corresponds to nearly an entire grazing season in northern latitudes of the U.S. These residues can be viewed as desirable when they directly reduce populations of pestiferous fly species. However, these residues also adversely affect beneficial species, whose activities accelerate dung pat degradation, facilitate nutrient cycling, and indirectly reduce populations of pestiferous flies and parasites affecting livestock. The implications of long term parasiticide excretion for pasture ecosystems merit careful consideration.

## Supporting information

S1 Methods Description of additional reagents for the determination of eprinomectin residues in dung. (PDF)

## References

[1] González Canga A, Sahagún Prieto AM, José Diez Liébana M, Martínez NF, Vega MS, Vieitez JJG. The pharmacokinetics and metabolism of ivermectin in domestic animal species. Vet J. 2009;179: 25–37. 10.1016/j.tvjl.2007.07.011 17851096

[2] Aksit D, Korkut O, Aksoz E, Gokbulut C. Plasma disposition and faecal excretion of eprinomectin following topical and subcutaneous administration in non-lactating dairy cattle. N Z Vet J. 2016;169: 1–16. 10.1080/00480169.2016.1146172 26820168

[3] Floate KD. Off-target effects of ivermectin on insects and on dung degradation in southern Alberta, Canada. Bull Entomol Res. 1998;88: 25–35.

[4] Floate KD, Bouchard P, Holroyd G, Poulin R, Wellicome TI. Does Doramectin Use on Cattle Indirectly Affect the Endangered Burrowing Owl. Rangel Ecol Manag. 2008;61: 543–553.

[5] Errouissi F, Alvinerie M, Galtier P, Kerboeuf D, Lumaret JP. The negative effects of the residues of ivermectin in cattle dung using a sustained-release bolus on *Aphodius constans* (Duft.) (Coleoptera: Aphodiidae). Vet Res. 2001;32: 421–427. 10.1051/vetres:2001134 11592612

[6] Fincher GT. Injectable ivermectin for cattle: Effects on some dung-inhabiting insects. Environ Entomol. 1992;21: 871–876.

[7] Iwasa M, Sugitani M. Effects of the veterinary antiparasitic drug eprinomectin on dung beetles (Coleoptera: Scarabaeidae), the non-pest fly *Neomyia cornicina* and pest fly *Haematobia irritans* (Diptera: Muscidae) in Japan. Appl Entomol Zool. 2014;49: 591–597.

[8] Römbke J, Barrett K, Blanckenhorn WU, Hargreaves T, Kadiri N, Knäbe S, et al Results of an international ring test with the dung fly *Musca autumnalis* in support of a new OECD test guideline. Sci Total Environ. 2010;408: 4102–4106. 10.1016/j.scitotenv.2010.05.027 20542534

[9] Floate KD, Colwell DD, Fox AS. Reductions of non-pest insects in dung of cattle treated with endectocides: a comparison of four products. Bull Entomol Res. 2002;92: 471–481. 17598298 10.1079/ber2002201

[10] Wright EJ, Muller P. Laboratory studies of host finding, acceptance and suitability of the dung-breeding fly, *Haematobia thirouxi potans* [Dipt.: Muscidae], by *Aleochara* sp. [Col.: Staphylinidae]. Entomophaga. 1989;34: 61–71.

[11] Greene GL. Occurrence of *Aleochara* spp. (Coleoptera: Staphylinidae) Parasitoidism of filth fly pupae in western Kansas. J Kans Entomol Soc. 1997;1: 70–72.

[12] Hu GY, Frank JH. Predation on the horn fly (Diptera: Muscidae) by five species of *Philonthus* (Coleoptera: Staphylinidae). Environ Entomol. 1997;26: 1240–1246.

[13] Anderson JR, Merritt RW, Loomis EC. The insect-free cattle dropping and its relationship to increased dung fouling of rangeland pastures. J Econ Entomol. 1984;77: 133–141.

[14] Nichols E, Spector S, Louzada J, Larsen T, Amezquita S, Favila ME. Ecological functions and ecosystem services provided by Scarabaeinae dung beetles. Biol Conserv. 2008;141: 1461–1474.

[15] Merial Ltd. LongRange Technical Manual. 2012. http://thelongrangelook.com/wp-content/uploads/2015/07/LONGRANGE_Tech_Manual.pdf Cited 18 June 2018.

[16] Gotelli NJ, Chao A. Measuring and estimating species richness, species diversity and biotic similarity from sampling data [Internet]. Encyclopedia of Biodiversity. Elsevier Ltd.; 2013.

[17] Floate KD, Düring RA, Hanafi J, Jud P, Lahr J, Lumaret JP, et al Validation of a standard field test method in four countries to assess the toxicity of residues in dung of cattle treated with veterinary medical products. Environ Toxicol Chem. 2016;35: 1934–1946. 10.1002/etc.3154 26174741

[18] Wohde M, Blanckenhorn WU, Floate KD, Lahr J, Lumaret JP, Römbke J, et al Analysis and dissipation of the antiparasitic agent ivermectin in cattle dung under different field conditions. Environ Toxicol Chem. 2016;35: 1924–1933. 10.1002/etc.3462 27100922

[19] Kožuh Eržen N, Hodošček L, Cerkvenik-Flajs V. Analytical procedure for determination of the time profile of eprinomectin excretion in sheep faeces. Anal and Bioanal Chem. 2007;387: 1329–1335. 17109134 10.1007/s00216-006-0903-6

[20] Team RC. R: A language and enviornment for statistical computing. R Foundation for Statistical Computing, Vienna, Austria 2016.

[21] Wardhaugh KG, Mahon RJ. Avermectin residues in sheep and cattle dung and their effects on dung-beetle (Coleoptera: Scarabaeidae) colonization and dung burial. Bull Entomol Res. 1991;81: 333–339.

[22] Holter P, Sommer C, Grønvold J, Madsen M. Effects of ivermectin treatment on the attraction of dung beetles (Coleoptera: Scarabaeidae and Hydrophilidae) to cow pats. Bull Entomol Res. 1993;83: 53–58.

[23] Lumaret JP, Galante E, Lumbreras C, Mena J, Bertrand M, Bernal JL, et al Field Effects of Ivermectin Residues on Dung Beetles. J Appl Ecol. 1993;1: 428–436.

[24] Floate KD. Does a repellent effect contribute to reduced levels of insect activity in dung from cattle treated with ivermectin? Bull Entomol Res. 1998;88: 291–297.

[25] Floate KD. Endectocide residues affect insect attraction to dung from treated cattle: Implications for toxicity tests. Med Vet Entomol. 2007;21: 312–322. 10.1111/j.1365-2915.2007.00702.x 18092969

[26] Errouissi F, Lumaret JP. Field effects of faecal residues from ivermectin slow-release boluses on the attractiveness of cattle dung to dung beetles. Med Vet Entomol. 2010;24: 433–440. 10.1111/j.1365-2915.2010.00891.x 20629952

[27] Webb L, Beaumont DJ, Nager RG, McCracken DI. Field-scale dispersal of Aphodius dung beetles (Coleoptera: Scarabaeidae) in response to avermectin treatments on pastured cattle. Bull Entomol Res. 2010;100: 175–183. 10.1017/S0007485309006981 19586576

[28] Scheffczyk A, Floate KD, Blanckenhorn WU, Düring RA, Klockner A, Lahr J, et al Nontarget effects of ivermectin residues on earthworms and springtails dwelling beneath dung of treated cattle in four countries. Environ Toxicol Chem. 2016;35: 1959–1969. 10.1002/etc.3306 26565894

[29] Wardhaugh KG, Longstaff BC, Morton R. A comparison of the development and survival of the dung beetle, *Onthophagus taurus* (Schreb.) when fed on the faeces of cattle treated with pour-on formulations of eprinomectin or moxidectin. Vet Parasitol. 2001;99: 155–168. 11470182 10.1016/s0304-4017(01)00451-4

[30] Lumaret JP, Errouissi F, Galtier P, Alvinerie M. Pour-on formulation of eprinomectin for cattle: Fecal elimination profile and effects on the development of the dung-inhabiting diptera *Neomyia cornicina* (L.) (Muscidae). Environ Toxicol Chem. 2005;24: 797–801. 15839552 10.1897/03-583.1

[31] Lumaret J-P, Errouissi F, Floate K, Römbke J, Wardhaugh K. A review on the toxicity and non-target effects of macrocyclic lactones in terrestrial and aquatic environments. Curr Pharm Biotechnol. 2012;13: 1004–1060. 10.2174/138920112800399257 22039795 PMC3409360

[32] National Academies of Sciences, Engineering, and Medicine. Nutrient requirements of beef cattle. National Academies Press; 2016 6 16.

