# Supplementary material for: Eprinomectin from a sustained release formulation adversely affected dung breeding insects
Source: PLoS One. 2018 Aug 6;13(8):e0201074. doi: 10.1371/journal.pone.0201074 (PMC6078490; doi:10.1371/journal.pone.0201074)
Supplement: S1 Methods — (PDF) [file pone.0201074.s001.pdf]

S1 Methods. Description of additional reagents for the determination of eprinomectin residues in dung.

N-methylimidazole (purity >99%), trifluoroacetic anhydride (purity >99%), and triethylamine (purity >99%) were provided by Sigma-Aldrich Chemie GmbH, and glacial acetic acid (100% EMSURE® anhydrous for analysis) by Merck KGaA. Purified water was taken from a Milli-Q® water purification system (Merck Millipore). Stock solutions and working standard solutions were prepared by dissolving 5 mg eprinomectin and ivermectin each in 5 mL acetonitrile and placing the solutions in an ultrasonic bath for 30 s; stock solutions were then stored at -32°C. With these, we prepared calibration standards for eprinomectin (0.5, 1, 2, 5, 10, 50, 100, 500 µg L^{-1}) and a 2000 µg L^{-1} working solution for ivermectin.

After extraction with acetonitrile, we diluted 10 mL of each extract with 33.3 mL purified water and 33.3 µL trimethylamine, and SPE cartridges were conditioned with 10 mL acetonitrile and 10 mL acetonitrile/water (3:10, v/v). Elution followed with 10 mL acetonitrile without a prior washing step, because during method development we found that polar matrix components did not interfere with the separation process. Eluates were evaporated to dryness under a gentle stream of nitrogen at 60°C and thereafter frozen for 24 to 96 h at -32°C until derivatization.

Derivatization for eprinomectin and the internal standard ivermectin was performed according to Kozuh Erzen et al. (2007) with following variations: 700 µL of processed extract were derivatized with 111 µL N-methylimidazole-acetonitrile (1:1, v/v), 167 µL trifluoroacetic anhydride-acetonitrile (1:1, v/v), and 133 µL glacial acetid acid. Eprinomectin calibration

standards were derivatized likewise with the prior addition of 250 µL ivermectin working solution and thus 1.36 times more of the three derivatization reagents. After adding these reagents, we continued the derivatization and followed with HPLC-fluorescence detection as described by Wohde et al. (2016) within 2 h after derivatization. The gradient elution was performed with a mobile phase of purified water (A) and acetonitrile (B) at a flow rate of 0.3 mL min^{-1}. The gradient was: 0 min to 10 min, 88% to 100% B; 10 min to 11 min 100% B. The injection volume was 40 µL. Excitation wavelength was 364 nm and emission wavelength 463 nm. Column temperature was 30°C on a Dionex Acclaim PolarAdvantage II C18-Column (150 mm, 2.1 mm inner diameter, 3 µm particle size). This highly selective analytical procedure allows unequivocal attribution of the fluorescence signal to eprinomectin and not transformation products.

Evaluation of the internal standard ivermectin in each sample gave recovery rates ranging from 90 to 116%, clearly indicating full recovery for ivermectin in the dung extracts. Results for eprinomectin analysis were not corrected for internal standard recovery rates. For eprinomectin, recovery rates were assessed (4 replicates) by spiking untreated dung from week 0 of the experiment to a concentration of 200 ng/g eprinomectin. After extraction and analysis corresponding to the above method a recovery rate of 74.5 % (SD 1.8 %, n=4) was determined. The limit of detection for eprinomectin in fresh dung was determined as 0.5 ng/g, the limit of quantification as 1.7 ng/g, expressed respectively as 3 and 10 times the signal to noise ratio. The process of extraction and measurement was performed in triplicate for each sampling day.
